# 

**Published:** 1845-08

**Authors:** 


CONTENTS:

[Review.]—Vital Chemistry—Lectures on Animal Heat. By THOS. SPENCER, M.D., Prof. of the Institutes and Prac. of Med., in the Med. Institution of Geneva College, - - - - 65

BIBLIOGRAPHICAL NOTICES.

Mental Maladies—A Treatise on Insanity. By E. ESQUIROL, Physician-in-Chief of the Maison Royale des Alienes de Charenton. Translated from the French, with additions, by E. K. Hunt, M. D. - - - - 70

MEDICAL INTELLIGENCE.

Medical Schools, - - - 72
Appointments, - - - 73
Salacine—the Sugeon General's Circular, - - - 74

PRACTICAL MEDICINE, &c.

Neuralgia—Introduction of Medicated Fluid to the Nerve. By Mr. RYND, - - - 75
Chemical Analysis of Rhubarb or Pie-plant. By Lieut. E. R. LONG, M. D., U. S. A., - - 78
On the frequent Spontaneous Cure of Pulmonary Consumption, and the Indications furnished by Pathology for its Rational Treatment, - - - - 79
Adulteration of Saffron, - - 80
Correction, - - 80
Erratum, - - - - 80

